# A Western‐style obesogenic diet alters maternal metabolic physiology with consequences for fetal nutrient acquisition in mice

**DOI:** 10.1113/JP273684

**Published:** 2017-04-05

**Authors:** Barbara Musial, Owen R. Vaughan, Denise S. Fernandez‐Twinn, Peter Voshol, Susan E. Ozanne, Abigail L. Fowden, Amanda N. Sferruzzi‐Perri

**Affiliations:** ^1^ Department of Physiology, Development and Neuroscience University of Cambridge Cambridge UK; ^2^ University of Cambridge Metabolic Research Laboratories, and MRC Metabolic Disease Unit, Wellcome Trust‐MRC Institute of Metabolic Science Addenbrooke's Hospital Cambridge UK

**Keywords:** fetal growth, gestational diabetes, glucose metabolism, insulin resistance, nutrient partitioning, pregnancy

## Abstract

**Key points:**

- In the Western world, obesogenic diets containing high fat and high sugar (HFHS) are commonly consumed during pregnancy, although their effects on the metabolism of the mother, in relation to feto‐placental glucose utilization and growth, are unknown.
- In the present study, the consumption of an obesogenic HFHS diet compromised maternal glucose tolerance and insulin sensitivity in late pregnancy in association with dysregulated lipid and glucose handling by the dam.
- These maternal metabolic changes induced by HFHS feeding were related to altered feto‐placental glucose metabolism and growth.
- A HFHS diet during pregnancy therefore causes maternal metabolic dysfunction with consequences for maternal nutrient allocation for fetal growth.
- These findings have implications for the health of women and their infants, who consume obesogenic diets during pregnancy.

**Abstract:**

In the Western world, obesogenic diets containing high fat and high sugar (HFHS) are commonly consumed during pregnancy. However, the impacts of a HFHS diet during pregnancy on maternal insulin sensitivity and signalling in relation to feto‐placental growth and glucose utilization are unknown. The present study examined the effects of a HFHS diet during mouse pregnancy on maternal glucose tolerance and insulin resistance, as well as, on feto‐placental glucose metabolism. Female mice were fed a control or HFHS diet from day (D) 1 of pregnancy (term = D20.5). At D16 or D19, dams were assessed for body composition, metabolite and hormone concentrations, tissue abundance of growth and metabolic signalling pathways, glucose tolerance and utilization and insulin sensitivity. HFHS feeding perturbed maternal insulin sensitivity in late pregnancy; hepatic insulin sensitivity was higher, whereas sensitivity of the skeletal muscle and white adipose tissue was lower in HFHS than control dams. These changes were accompanied by increased adiposity and reduced glucose production and glucose tolerance of HFHS dams. The HFHS diet also disturbed the hormone and metabolite milieu and altered expression of growth and metabolic signalling pathways in maternal tissues. Furthermore, HFHS feeding was associated with impaired feto‐placental glucose metabolism and growth. A HFHS diet during pregnancy therefore causes maternal metabolic dysfunction with consequences for maternal nutrient allocation for fetal growth. These findings have implications for the health of women and their infants, who consume HFHS diets during pregnancy.

## Key points

- In the Western world, obesogenic diets containing high fat and high sugar (HFHS) are commonly consumed during pregnancy, although their effects on the metabolism of the mother, in relation to feto‐placental glucose utilization and growth, are unknown.
- In the present study, the consumption of an obesogenic HFHS diet compromised maternal glucose tolerance and insulin sensitivity in late pregnancy in association with dysregulated lipid and glucose handling by the dam.
- These maternal metabolic changes induced by HFHS feeding were related to altered feto‐placental glucose metabolism and growth.
- A HFHS diet during pregnancy therefore causes maternal metabolic dysfunction with consequences for maternal nutrient allocation for fetal growth.
- These findings have implications for the health of women and their infants, who consume obesogenic diets during pregnancy.

**Abbreviations**
AUC: area under the curve
2DG: ^14^C‐2‐deoxy‐glucose
D: day
EGP: endogenous glucose production
G6Pase: glucose 6‐phosphatase
GDM: gestational diabetes
GIR: glucose infusion rate
HEC: hyperinsulinaemic‐euglycaemic clamp
HFHS: high fat and high sugar
IGF1: insulin‐like growth factor‐1
NEFA: non‐esterified free fatty acid
PEPCK: phosphoenolpyruvate carboxykinase
PI3K: phosphoinositol 3‐kinase
*R*_a_: rate of appearance
*R*_d_: rate of disappearance
TG: triglyceride
WAT: white adipose tissue

## Introduction

In the developed Western world, women of child‐bearing age obtain a high proportion of their energy intake from fat and/or sugar (Balanza *et al*. 2007; Temme *et al*. 2010; Austin *et al*. 2011). These dietary habits are contributing to the increasing number of women who gain excess weight during pregnancy and/or develop gestational diabetes (GDM); a condition affecting 5–10% of pregnant women (Schieve *et al*. 1998; Agha‐Jaffar *et al*. 2016; Koning *et al*. 2016). Increased maternal fat deposition and/or obesity during pregnancy are also associated with health complications for the mother and infant after birth (Thorsdottir *et al*. 2002; Berggren *et al*. 2016). Mothers gaining excess weight during pregnancy have a greater probablility of retaining the weight post‐delivery and are at increased risk of obesity, type‐2 diabetes and cardiovascular disease later in life (Frias & Grove, 2012; Berggren *et al*. 2016). Their infants are also often of abnormal birth weight and more prone to obesity, hypertension and type‐2 diabetes as adults (Thorsdottir *et al*. 2002; Oken *et al*. 2007; Frias & Grove, 2012). Experimental animal studies have supported the human observational studies and shown that feeding diets high in fat and/or sugar during pregnancy programs metabolic dysfunction in the adult offspring (Khan *et al*. 2005; Samuelsson *et al*. 2008; Elahi *et al*. 2009; Ashino *et al*. 2012; Franco *et al*. 2012; Alfaradhi *et al*. 2014; Fernandez‐Twinn *et al*. 2014). However, the mechanisms operating during pregnancy that mediate the programming effects of these diets on the mother and her offspring remain largely unknown.

To sustain pregnancy and fetal growth, nutrients must be appropriately partitioned between the gravid uterus and mother. To achieve this, the mother's metabolism is shifted to a more insulin resistant state such that her own glucose utilization is limited, in favour of fetal supply (Butte, 2000; Catalano *et al*. 2002; Musial *et al*. 2016). Whole body insulin resistance during pregnancy results from changes in the action of insulin in the liver, skeletal muscle and adipose tissue. These adaptations are assumed to be signalled, in part by changes in placental hormone production. In non‐pregnant animals, insulin resistance and altered insulin signalling in these tissues are observed in response to feeding Western‐style diets high in fat and sugar (HFHS) (Um *et al*. 2004; Wang *et al*. 2015; Zhang *et al*. 2015). However, the effects of feeding HFHS diets during pregnancy on insulin sensitivity and tissue insulin signalling pathways in the pregnant mother remain unknown. Furthermore, it is not clear whether feto‐placental glucose utilization is altered in these circumstances. Therefore, the present study aimed to examine the effects of a HFHS diet during mouse pregnancy on maternal glucose tolerance and insulin resistance and on feto‐placental glucose metabolism. It also determined the effect of HFHS feeding on placental expression of prolactin/placental lactogen (*Prl/Pl*) related genes, which change with gestational age and have proposed metabolic effects in the mother (Simmons *et al*. 2008).

## Methods

### Animals

All experiments were carried out under the UK Home Office Animals (Scientific Procedures) Act 1986, following ethical review by the University of Cambridge. C57Bl/6 females were housed under a 12:12 h dark/light photocycle with free access to water and a standard rodent diet (RM3; Special Dietary Services, Witham, UK). At 8–12 weeks of age, 92 females were mated with C57Bl/6 males and the day of copulatory plug detection was defined as day (D)1 of pregnancy. From D1, females were maintained on either the standard diet (*n* = 50) or were fed a HFHS diet (*n* = 42). The nutritional composition of both diets has been reported previously (Sferruzzi‐Perri *et al*. 2013). All procedures were performed on either D16 or D19 of pregnancy and mice were allocated for tissue and blood collection, a glucose tolerance test or a hyperinsulinaemic‐euglycaemic clamp.

### Tissue and blood collection

Between 08.00 and 10.00 h, unfasted pregnant dams (*n* = 10 per diet, per age) were weighed and anaesthetized (10 μl g^−1^ fentanyl‐fluanisone:midazolam in sterile water, 1:1:2; i.p.; Janssen Animal Health, Beerse, Belgium). A cardiac blood sample was taken before death by cervical dislocation. Liver and retroperitoneal white adipose tissue (WAT) were dissected, weighed and snap frozen in liquid nitrogen for western blotting. Maternal skeletal muscle (biceps femoris) was also dissected and snap frozen. Blood glucose concentrations were measured using a hand‐held glucometer (One Touch Ultra; LifeScan, Johnson & Johnson Medical Ltd, Livingston, UK). At D19, the maternal liver was also fixed in 4% paraformaldehyde, dehydrated and embedded into paraffin wax before sectioning at 8 μm and staining with haematoxylin and eosin. Tissues and blood taken from dams in the previously published cohort (Sferruzzi‐Perri *et al*. 2013), contributed to the samples used for biometrical and biochemical analyses on the mother and placental gene expression.

### Glucose tolerance test (GTT)

Conscious dams underwent a GTT on D16 or D19 (*n* = 6–9 per diet, per age) after fasting from 08.00 h for 6 h. Blood samples (≤5 μl) were taken from the tail vein immediately before i.p. glucose administration (10% w/v, 1 g kg^−1^ body weight) and, subsequently, at 15, 30, 45, 60, 90 and 120 min to measure glucose concentrations as above. Mice were then killed by cervical dislocation.

### Hyperinsulinaemic‐euglycaemic clamp (HEC)

The HEC was performed as described previously (Musial *et al*. 2016) on D19 mice. Mice fasted for 2.5 h (*n* = 6 per diet) were anaesthetized with a mixture of ventranquil:dormicum:fentanyl (1:2:10 in 3 units of water, 10 μl g^−1^ body weight, i.p., Janssen‐Cilag, Tilburg, The Netherlands) and maintained at 37°C using a servo‐controlled thermopad (Harvard Instruments, Cambournse, UK). After catheterizing a tail vein, d‐^3^H‐glucose was infused continuously (0.006 MBq min^−1^ in PBS, 50 μl h^−1^, i.v., 370–740 GBq mmol^−1^; PerkinElmer, Wokingham, UK). After steady‐state was achieved at 60 min (basal state, ∼3.5 h fasted), two blood samples were taken 10 min apart. Insulin was then injected as a bolus (3.3 mU, i.v.; human insulin; Actrapid; Novo Nordisk, Bagsværd, Denmark) followed by infusion (0.09 mU min^−1^) together with the d‐^3^H‐glucose. Blood glucose concentrations were monitored throughout the protocol and a variable rate glucose infusion (12.5% w/v PBS, Sigma, i.v.) was begun 10 min later to maintain blood glucose at mean basal levels. At 50 min after insulin administration, 2‐deoxy‐glucose (^14^C‐2DG, specific activity: 9.25–13.0 GBq mmol^−1^; PerkinElmer) was injected i.v. By 70 min of insulin administration, blood glucose levels were clamped at basal concentrations and a further three blood samples were collected at 10 min intervals. Mice were then killed by cervical dislocation and samples of biceps femoris and white adipose tissue were collected together with the fetus and placenta adjacent to the cervix in each horn (*n* = 2 fetuses and placentas per litter) for analysis of tissue ^14^C‐2DG content. Rates of glucose utilization and production and whole body and hepatic insulin sensitivity were calculated as described previously (Voshol *et al*. 2001).

### Hormone and metabolite concentrations

Plasma d‐^3^H‐glucose was measured by scintillation counting (Hidex 300SL; LabLogic Ltd, Sheffield, UK), plasma leptin, insulin, triglycerides (TG), cholesterol, non‐esterified free fatty acids (NEFA) and insulin‐like growth factor‐1 (IGF1) concentrations determined by immuno and enzymatic assays (Musial *et al*. 2016), and plasma corticosterone measured by radioimmunoassay (MP Biomedicals, Carlsbad, CA, USA) (Sferruzzi‐Perri *et al*. 2011). Plasma insulin concentrations during the HEC were measured by ELISA (Crystal Chem, Inc., Downer's Grove, IL, USA), which detected both mouse and human (Musial *et al*. 2016).

### Tissue biochemical composition

Hepatic glycogen content was measured enzymatically using amyloglucosidase as reported previously (Roehrig & Allred, 1974). The fat content of the liver and pooled samples of skeletal muscle were measured using the modified Folch method (Folch *et al*. 1957). To determine tissue phosphorylated 2DG (p2DG) content, maternal tissues and whole fetuses were homogenized in 0.5% perochloric acid and the homogenates neutralized to separate p2DG from 2DG by precipitation before scintillation counting, as described previously (Musial *et al*. 2016).

### Tissue protein expression

Proteins were extracted (∼50–100 mg, *n* = 5–6 per diet per age) from the maternal liver, skeletal muscle and WAT for assessment of insulin signalling and lipid metabolism pathways using western blotting as described previously (Musial *et al*. 2016). In addition, phosphoenolpyruvate carboxykinase (PEPCK) and glucose 6‐phosphatase (G6Pase) were detected using antibodies from Santa Cruz Biotechnology (Santa Cruz, CA, USA) (sc‐32789 and sc‐27198, respectively). Protein bands were quantified using ImageJ (National Institutes of Health, Bethesda, MD, USA). To control for variations in loading, expression of the proteins of interest was normalized to a corresponding band of similar molecular weight on the ponceau S‐stained membrane.

### Placental gene expression

RNA was extracted from placentas with the second lightest weight for the litter using the RNeasy Plus Mini Kit (Qiagen, Manchester, UK). RNA was then reverse transcribed using Multiscribe Reverse Transcriptase with random primers (Applied Biosystems, Foster City, CA, USA) and analysed in duplicate using Taqman assays for *Prl/Pl*‐related genes, *Prl5a1*, *Prl2b1*, *Prl7b1*, *Prl7a2*, *Prl3d1* and *Prl3b1* (Mm00517851_m1, Mm01163874_m1, Mm01164616_m1, Mm00478316_m1, Mm04213281_m1 and Mm00435852_m1, respectively) and the housekeepers *Hprt* and *Gadph* (Mm00446968 and Mm99999915_g1, respectively). The genes of interest were normalized to the geometric mean expression levels of the housekeepers, which remained constant across diets and age. Data were analysed using the 2^–ΔΔCT^ method (Sferruzzi‐Perri *et al*. 2013).

### Statistical analysis

Data are reported as the mean ± SEM. All statistical analyses and calculations were performed using Prism (GraphPad Software Inc., San Diego, CA, USA). The effect of diet at different ages was assessed using an unpaired *t* test. Changes within the same dietary group were assessed by a paired *t* test. In the GTT protocol, the changes in glucose concentration were analysed by two‐way ANOVA with time as a repeated measure and the area under the curve (AUC) calculated using the trapezoid rule. *P* < 0.05 was considered statistically sigificant.

## Results

### Biometry

On D16 of pregnancy, whole body but not hysterectomized weight was lower for HFHS compared to control dams (Table 1). HFHS dams had a lighter liver but greater white adipose tissue (retroperitoneal fat, WAT) mass compared to control dams on D16. There were no significant changes in maternal biometry with diet at D19 (Table 2). HFHS diet reduced both fetal and placental weight on D16, whereas only placental weight remained significantly decreased on D19 (Tables 1 and 2). There was no effect of diet at either time point on the ratio of fetal to placental weight per litter, nor any affect on litter size (Tables 1 and 2).

**Table 1:** The effect of HFHS feeding on maternal hormone and metabolite concentrations and biochemical composition of liver, skeletal muscle and white adipose tissue at day 16 of pregnancy

	Control	HFHS
Tissue weights		
Maternal whole body (g)	31.4 ± 1.1	29.0 ± 0.5*
Maternal hysterectomized (HW, g)	24.1 ± 0.7	22.6 ± 0.4
Maternal liver (g)	1.90 ± 0.06	1.68 ± 0.05*
Maternal liver (% HW)	7.9 ± 0.1	7.5 ± 0.2
Maternal WAT (g)	0.08 ± 0.01	0.11 ± 0.01*
Maternal WAT (% HW)	0.31 ± 0.03	0.46 ± 0.04*
Average fetus for litter (mg)	427 ± 12	394 ± 5*
Average placenta for litter (mg)	104 ± 2	94 ± 2*
Total fetal mass/total placental mass per litter	4.1 ± 0.1	4.2 ± 0.1
Viable pups per litter	7.5 ± 0.5	7.0 ± 0.4
Hormones and metabolites		
Blood glucose fed state (mm)	10.3 ± 0.3	11.8 ± 0.6*
Fasted 6 h (mm)	6.8 ± 0.2	7.6 ± 0.4*
Plasma NEFA (μm)	256 ± 42	256 ± 29
Plasma TG (mm)	1.2 ± 0.2	0.8 ± 0.1*
Plasma cholesterol (mm)	1.4 ± 0.1	2.2 ± 0.1*
Plasma insulin (μg l^–1^)	1.3 ± 0.3	2.8 ± 0.5*
Plasma leptin (pg ml^–1^)	4058 ± 656	13835 ± 2095*
Plasma IGF1 (pg ml^–1^)	299 ± 23	193 ± 29*
Plasma corticosterone (ng ml^–1^)	852 ± 160	849 ± 100
Tissue composition		
*Liver*		
Protein content (mg g^–1^)	177 ± 7.0	187 ± 13
Glycogen (mg g^–1^)	49 ± 1.1	54 ± 6.0
Total glycogen (mg)	86 ± 3.3	90 ± 11.9
Fat content (%)	5.1 ± 0.3	6.4 ± 0.8
Total fat content (mg)	91 ± 6.1	105 ± 12.8
*Skeletal muscle*		
Protein content (mg g^–1^)	71 ± 4.3	73 ± 8.0
Fat content (%)^†^	15	15
*WAT*		
Protein content (mg g^–1^)	27 ± 3.5	14 ± 1.0*

Data are from dam in fed state, unless otherwise indicated. Data are expressed as the mean ± SEM with 11–15 dams per diet, 5–16 per diet for plasma analysis and 5 or 6 dams per diet for biochemical composition. The effect of the diet was analysed by an unpaired *t* test. ^*^Significant difference from control diet (*P* < 0.05). ^†^Skeletal muscle fat content was measured on samples pooled from 4 or 5 animals from each experimental group. HW, hysterectomized weight.

**Table 2:** The effect of HFHS feeding on maternal hormone and metabolite concentrations and biochemical composition of liver, skeletal muscle and white adipose tissue at day 19 of pregnancy

	Control	HFHS
Tissue weights		
Maternal whole body (g)	35.7 ± 1.0	35.1 ± 0.8
Maternal hysterectomized (HW, g)	24.0 ± 0.6	23.1 ± 0.4
Maternal liver (g)	1.86 ± 0.07	1.68 ± 0.06
Maternal liver (% HW)	8.0 ± 0.3	7.4 ± 0.2
Maternal WAT (g)	0.06 ± 0.01	0.08 ± 0.01
Maternal WAT (% HW)	0.27 ± 0.03	0.35 ± 0.03
Average fetus for litter (mg)	1200 ± 14	1184 ± 18
Average placenta for litter (mg)	89 ± 2	82 ± 2*
Total fetal mass/total placental mass per litter	13.6 ± 0.3	14.4 ± 0.4
Viable pups per litter	7.3 ± 0.4	7.6 ± 0.4
Hormones and metabolites		
Blood glucose fed state (mm)	9.4 ± 0.6	10.2 ± 0.6
Fasted 6 h (mm)	5.2 ± 0.3	5.2 ± 0.3
Plasma NEFA (μm)	381 ± 41	225 ± 38*
Plasma TG (mm)	1.0 ± 0.1	0.7 ± 0.1
Plasma cholesterol (mm)	1.0 ± 0.1	1.2 ± 0.1
Plasma insulin (μg l^–1^)	0.6 ± 0.1	1.2 ± 0.5
Plasma leptin (pg ml^–1^)	3927 ± 709	5648 ± 1078
Plasma IGF1 (pg ml^–1^)	253 ± 20	205 ± 29
Plasma corticosterone (ng ml^–1^)	797 ± 115	750 ± 82
Tissue composition		
*Liver*		
Protein content (mg g^–1^)	186 ± 5.8	177 ± 4.6
Glycogen (mg g^–1^)	57 ± 6.2	55 ± 3.3
Total glycogen (mg)	103 ± 10.6	87 ± 8.8
Fat content (%)	4.7 ± 0.2	8.6 ± 0.8*
Total fat content (mg)	86 ± 5.9	138 ± 17.7*
*Skeletal muscle*		
Protein content (mg g^–1^)	74 ± 5.0	57 ± 6.1*
Fat content (%)^†^	20	31
*WAT*		
Protein content (mg g^–1^)	28 ± 3.0	18 ± 3.3*

Data are from dam in fed state, unless otherwise indicated. Data are expressed as the mean ± SEM with 12–22 dams for tissue weights, 5–16 per diet for plasma analysis and 5 or 6 dams per diet for biochemical composition. The effect of the diet was analysed by unpaired *t* test. ^*^Significant difference from control diet (*P* < 0.05). ^†^Skeletal muscle fat content was measured on samples pooled from 4 or 5 animals from each experimental group. HW, hysterectomized weight.

### Metabolites and hormone concentrations

In the fed state, HFHS dams were hyperglycaemic, hyperinsulinaemic, hyperleptinaemic and hypercholesterolaemic at D16 but not at D19 (Tables 1 and 2). They remained hyperglycaemic in the fasted state at D16 (Table 1). Plasma TG and IGF1 concentrations were lower in HFHS compared to control dams at D16 but not at D19 (Tables 1 and 2). Plasma NEFAs were unaffected at D16 but were decreased at D19 by HFHS feeding (Tables 1 and 2). There was no effect of a HFHS diet on maternal plasma corticosterone concentrations on either D16 or D19 of pregnancy (Tables 1 and 2).

### Tissue biochemical composition

**Figure 1:**
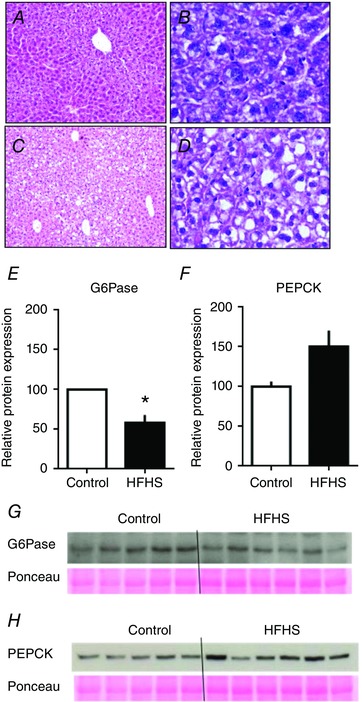
The effect of HFHS feeding on maternal hepatic fat accumulation and glucose handling on day 19 of pregnancy Representative photomicrographs of haematoxylin and eosin‐stained liver sections from dams at D19 that were fed a control (*A* and *B*) or HFHS diet (*C* and *D*). Photos taken under 10× (*A* and *C*) and 40× objective lens (*B* and *D*). Hepatic abundance of G6Pase (*E* and *G*) and PEPCK (*F* and *H*) in control (*n* = 5) and HFHS dams (*n* = 6) at D19. ^*^ *P* < 0.05. [Color figure can be viewed at wileyonlinelibrary.com]

Hepatic protein and glycogen content did not differ with diet at either stage of pregnancy (Tables 1 and 2). Hepatic fat content was higher in HFHS compared to control dams at D19 but not at D16, when expressed both as total weight and as a proportion of liver weight (Tables 1 and 2). Histological staining confirmed intrahepatic fat accumulation in D19 HSHF‐fed dams (Fig. 1 *A*–*D*). In skeletal muscle, protein content was lower, whereas fat content tended to be higher in HFHS compared to control dams at D19 only (Table 2). Protein content of WAT was lower in HFHS compared to control dams at both ages (Tables 1 and 2).

### Glucose tolerance

**Figure 2:**
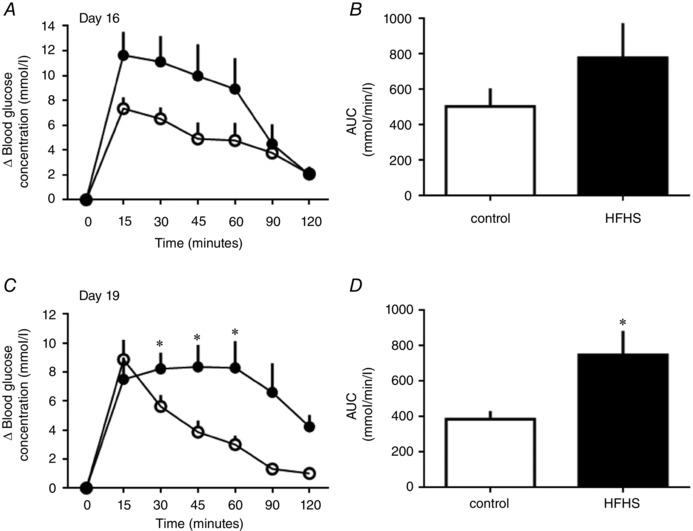
The effect of HFHS feeding on maternal glucose tolerance in late pregnancy Glucose tolerance in dams at D16 (*A* and *B*) and D19 (*C* and *D*) of pregnancy fed control (white symbols and columns, *n* = 8–9) or HFHS diets (black symbols or columns, *n* = 6–7). Changes in blood glucose concentrations from basal pre‐injection values with time after glucose administration (*A* and *C*) and AUC (*B* and *D*). ^*^ *P* < 0.05.

There was no significant difference in the increment in blood glucose or in the glucose AUC with diet at D16, although glucose concentrations appeared to be higher and remain elevated for longer in HFHS dams (Fig. 2 *A* and *B*). At D19, the glucose increment in HFHS dams was greater at 30, 45 and 60 min compared to control values (Fig. 2 *C*). Consequently, HSHF feeding led to a greater AUC (Fig. 2 *D*), indicative of diet‐induced glucose intolerance in late pregnancy.

### Insulin sensitivity

**Figure 3:**
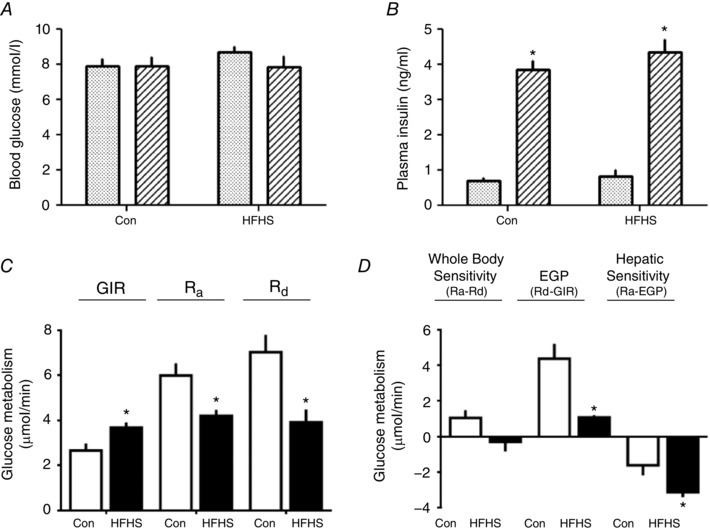
The effect of HFHS feeding on maternal glucose‐insulin dynamics on day 19 of pregnancy Blood glucose (*A*) and plasma insulin concentrations (*B*) in the basal (stippled columns) and hyperinsulinaemic (striped columns) periods of the hyperinsulinaemic‐euglycaemic clamp, rates of glucose infusion (GIR), appearance (*R*_a_) and disappearance (*R*_d_) measured directly (*C*) or derived indirectly as differences in rates (*D*) from tritiated glucose infusion and the clamp protocol (whole body and hepatic insulin sensitivity and EGP) in control dams (Con, *n* = 6, white columns) and HFHS (*n* = 6, black columns) at D19 of pregnancy. ^*^Significant difference in (*B*) in concentration from the basal period (*P* < 0.01, paired *t* test), within each period of the clamp. ^*^Significant differences in (*C*) and (*D*) in rates between diets (*P* < 0.05, unpaired *t* test).

Whole body and hepatic insulin sensitivity were examined by HEC on D19. In both groups, blood glucose concentrations were clamped at basal, euglycaemic levels by 70–90 min after beginning insulin infusion (Fig. 3 *A*). At this time, plasma insulin concentrations were within the post‐prandial range and three‐ to four‐fold higher than basal values in both groups (Fig. 3 *B*). Whole body insulin sensitivity, measured as the glucose infusion rate (GIR), was higher in HFHS compared to control dams (Fig. 3 *C*). However, when whole body insulin sensitivity was calculated as the difference between glucose utilization in hyperinsulinaemic (*R*_d_) and basal states (*R*_a_), neither group appeared to be insulin sensitive (paired *t* test, *P* > 0.05 for the difference, both groups) (Fig. 3 *D*). Endogenous glucose production (EGP) occurred in basal (*R*_a_) and hyperglycaemic states (*R*_d_ minus GIR) irrespective of diet (single *t* test greater than zero, *P* < 0.05 for all cases) but was lower with respect to rate in HFHS compared to control dams in both states (Fig. 3 *C* and *D*). Hepatic insulin sensitivity, calculated as a significant decrease in EPG between basal and hyperinsulinaemic states, was detected in both dietary groups (paired *t* test, *P* < 0.05) and was greater in HFHS compared to control dams (Fig. 3 *D*). In the basal fed state, G6Pase expression was reduced and PEPCK was not significantly altered In the liver of D19 HFHS‐fed dams (Fig. 1 *E*–*H*).

### Whole body and tissue glucose utilization

Whole body glucose utilization was less in HFHS compared to control dams, in both basal (*R*_a_) and hyperinsulinaemic (*R*_d_) conditions (Fig. 3 *C*). During hyperinsulinaemia, tissue glucose utilization, measured as p2DG content, was lower in skeletal muscle of HFHS compared to control dams but was unaffected by diet in adipose tissue (Table 3). Fetal but not placental p2DG content was also lower in HFHS compared to control dams (Table 3).

**Table 3:** The effect of HFHS feeding on glucose utilization by skeletal muscle and adipose tissue of pregnant mice and by the feto‐placental tissues at day 19

p2DG content (nmol mg^–1^)		
	Control	HFHS
Skeletal muscle	2.03 ± 0.53	0.51 ± 0.49*
White adipose tissue	1.33 ± 0.83	0.97 ± 0.43
Placenta	5.38 ± 1.16	3.81 ± 0.98
Fetus	1.76 ± 0.31	0.73 ± 0.17*

Data are expressed as the mean ± SEM for control (*n* = 6) and HFHS (*n* = 5–6). ^*^Significant difference from control diet (*P* < 0.05, unpaired *t* test).

### Maternal tissue insulin/IGF1 signalling and lipid metabolism

#### Liver

**Figure 4:**
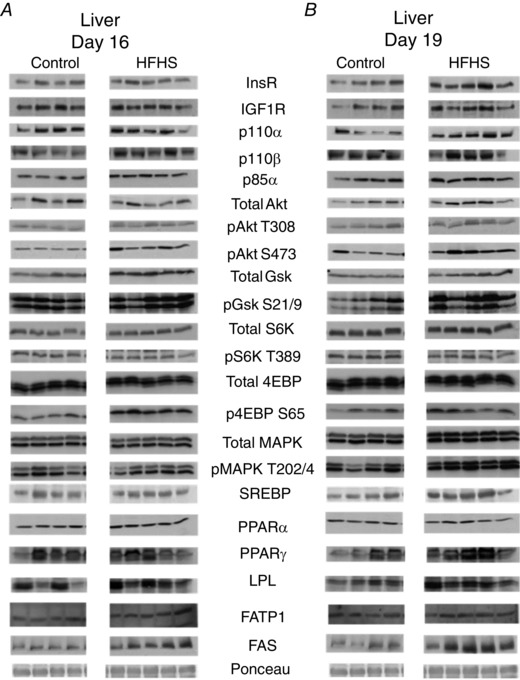
The effect of HFHS feeding on metabolic signalling pathways in the maternal liver in late pregnancy Western blots showing the abundance of insulin signalling and lipid metabolism proteins in the liver of dams at D16 (*A*) and D19 (*B*) of pregnancy fed control or HFHS diets.

**Figure 5:**
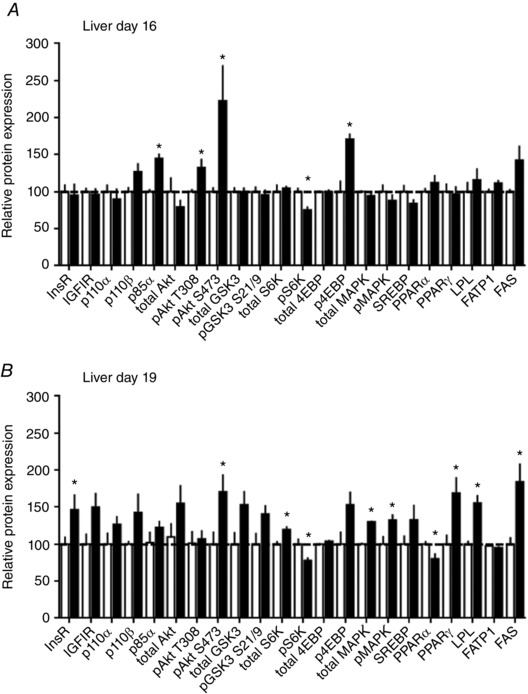
The effect of HFHS feeding on the expression of metabolic signalling pathways in the maternal liver in late pregnancy Quantification of insulin signalling and lipid metabolism proteins in liver of dams at D16 (*A*) and D19 (*B*) of pregnancy fed control (*n* = 5–6, white columns) or HFHS diets (*n* = 5–6, black columns). ^*^ *P* < 0.05.

The hepatic insulin/IGF1 signalling pathway was stimulated by HFHS feeding (Figs 4 and 5). At D16, p85α, pAkt T308 and pAktS473 were increased (Fig. 5 *A*). By D19, pAktS473 remained upregulated and the insulin receptor was also increased by HSHF feeding (Fig. 5 *B*). The mTORC1 signalling was disrupted by HFHS diet at both ages; pS6K was reduced and p4EBP increased at D16 and the total abundance of S6K was elevated although pS6K remained lower at D19, in the HFHS‐fed dams compared to controls (Fig. 5). MAPK signalling and lipid metabolism markers were unaffected by a HSHF diet at D16 (Fig. 5 *A*). At D19, total and phosphorylated MAPK were increased in HFHS dams (Fig. 5 *B*). Expression of lipogenic PPARγ, FAS and LPL was higher, whereas the lipid oxidation protein, PPARα was lower in HFHS compared to control dams (Fig. 5 *B*).

#### Skeletal muscle

**Figure 6:**
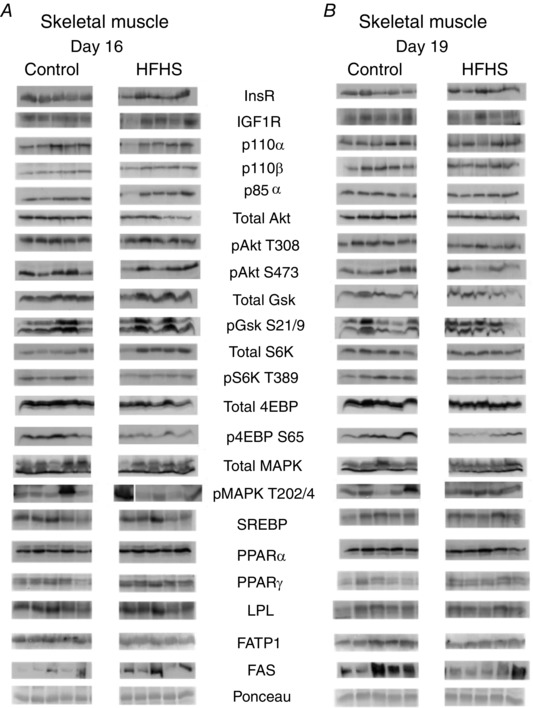
The effect of HFHS feeding on metabolic signalling pathways in the maternal skeletal muscle in late pregnancy Western blots showing the abundance of insulin signalling and lipid metabolism proteins in the skeletal muscle of dams at D16 (*A*) and D19 (*B*) of pregnancy fed control or HFHS diets.

**Figure 7:**
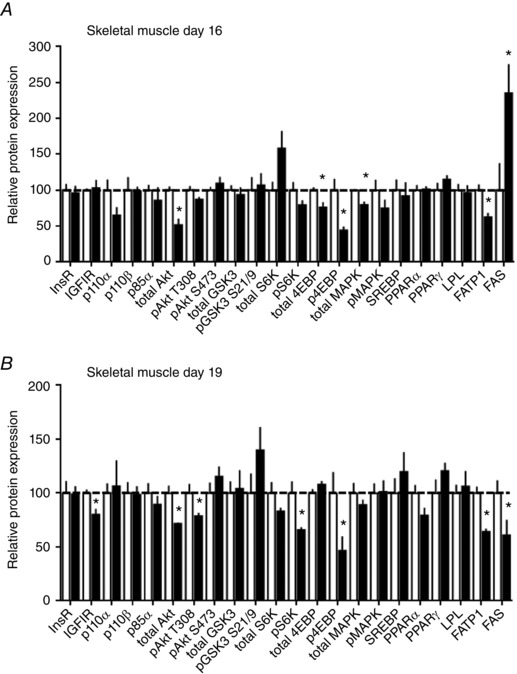
The effect of HFHS feeding on the expression of metabolic signalling pathways in the maternal skeletal muscle in late pregnancy Quantification of insulin signalling and lipid metabolism proteins in skeletal muscle of dams at D16 (*A*) and D19 (*B*) of pregnancy fed control (*n* = 5–6, white columns) or HFHS diets (*n* = 5–6, black columns). ^*^ *P* < 0.05.

The insulin/IGF1 signalling pathway was downregulated in the skeletal muscle by HFHS feeding at both D16 (total Akt, total MAPK, total 4EBP and p4EBP) and D19 (insulin‐like growth factor receptor 1 (IGF1R), total Akt, pAkt T308, pS6K and p4EBP) (Figs 6 and 7). Abundance of FATP1 was lower at both ages and FAS expression higher at D16 but reduced on D19 in HFHS compared to control dams (Fig. 7). The other markers of lipid metabolism assessed were unaffected by diet (Figs 6 and 7).

#### White adipose tissue

**Figure 8:**
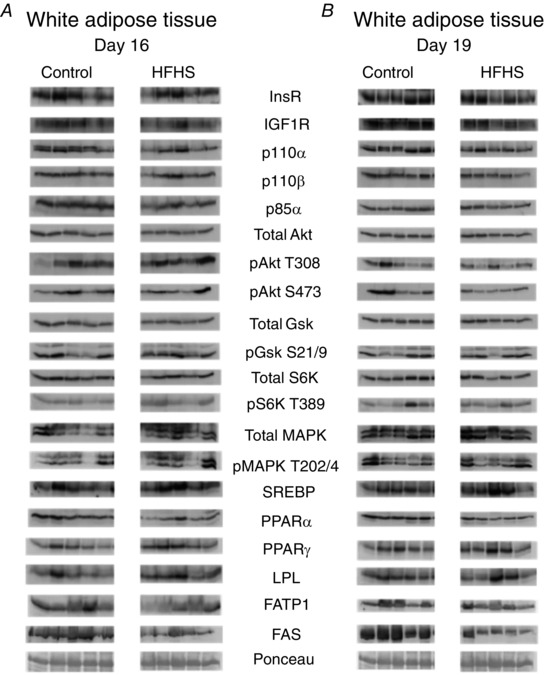
The effect of HFHS feeding on metabolic signalling pathways in the maternal white adipose tissue in late pregnancy Western blots showing the abundance of insulin signalling and lipid metabolism proteins in the white adipose tissue of dams at D16 (*A*) and D19 (*B*) of pregnancy fed control or HFHS diets.

**Figure 9:**
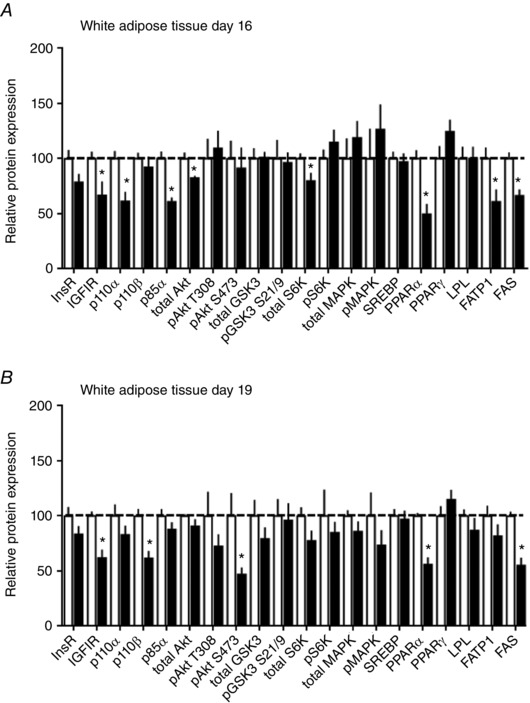
The effect of HFHS feeding on the expression of metabolic signalling pathways in the maternal white adipose tissue in late pregnancy Quantification of insulin signalling and lipid metabolism proteins in white adipose tissue of dams at D16 (*A*) and D19 (*B*) of pregnancy fed control (*n* = 5–6, white columns) or HFHS diets (*n* = 5–6, black columns). ^*^ *P* < 0.05.

HFHS feeding reduced the insulin/IGF1 signalling pathway in WAT (Figs 8 and 9). Expression of IGFIR, p110α, p85α, total Akt and total S6K was lower in HFHS compared to control dams at D16 (Fig. 9 *A*). At D19, IGFIR remained downregulated and p110β and pAkt S473 additionally reduced in HFHS‐fed compared to control dams (Fig. 9 *B*). WAT expression of FATP1 was decreased at D16 but normalized by D19, and PPARα and FAS were decreased at both ages in HFHS compared to control dams (Fig. 9). 4EBP could not be detected in WAT. The other marker of mTORC1 signalling, S6K, and MAPK signalling were unaltered by HSHF feeding (Figs 8 and 9).

#### Placental *Prl/Pl* gene expression

**Figure 10:**
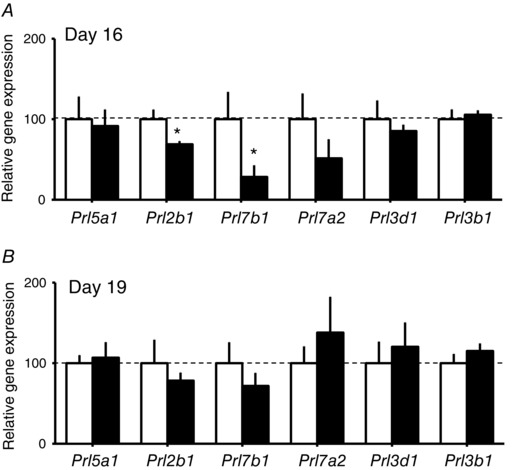
The effect of HFHS feeding on placental hormone expression in late pregnancy Expression of prolactins/placental lactogen‐related genes by the placenta on D16 (*A*) and D19 (*B*) of pregnancy in dams fed a control (*n* > 6, white columns) or HFHS diets (*n* > 6, black columns). ^*^ *P* < 0.05.

Placental expression of *Prl2b1* and *Prl7b1* genes was decreased in the placenta at D16 but normalized by D19 in HFHS compared to control dams (Fig. 10). There was no effect of HFHS feeding on the expression of *Prl5a1*, *Prl7a2*, *Prl3d1*, *Prl3b1* genes at either age.

## Discussion

The present study shows that excessive consumption of fat and sugar, typical of a Western‐style diet compromises maternal glucose tolerance and insulin sensitivity during late mouse pregnancy. These changes were accompanied by dysregulated lipid and glucose handling, a disturbed hormone and metabolite milieu, and altered expression of growth and metabolic signalling pathways in the liver, skeletal muscle and adipose tissue of the mother. Moreover, the maternal metabolic changes induced by HFHS feeding were related to altered feto‐placental glucose metabolism and growth, as well as *Prl/Pl*‐related gene expression in the placenta. A HFHS diet during pregnancy therefore, causes maternal metabolic dysfunction with consequences for maternal nutrient allocation for fetal growth. These findings have implications for the health of women and their infants in the Western world where HFHS diets are common during pregnancy.

The HFHS diet resulted in increased maternal adiposity during pregnancy; there was an expansion of WAT mass (significant at D16) and accumulation of fat in the liver and skeletal muscle at D19. Consistent with this, hepatic, skeletal muscle and WAT expression of lipogenic proteins were upregulated and/or lipid oxidation markers downregulated in HFHS dams at D19. Hepatic steatosis has previously been reported in pregnant rodents fed high fructose or high‐fat diets (Zou *et al*. 2012; Heerwagen *et al*. 2013). However, in the dams of the present study, the abundance of some lipogenic proteins was decreased, which may be a compensatory response to prevent further lipid accumulation in maternal tissues with HFHS feeding. In the skeletal muscle and WAT, the accumulation of lipids came at the expense of tissue protein. This was in line with diminished signalling via the protein synthesis pathway, mTORC1, in these tissues, as well as the increased whole body adiposity but reduced lean mass and dietary protein intake reported previously for dams on this HFHS diet (Sferruzzi‐Perri *et al*. 2013). The HFHS diet also affected conceptus mass, with a reduced weight of the fetus and placenta on D16 and a reduced placental weight on D19, as reported previously (Sferruzzi‐Perri *et al*. 2013). Taken together, these data suggest a global shift in nutrient partitioning between the dam and gravid uterus with HFHS feeding.

The HFHS diet also led to abnormal metabolite and hormone concentrations in the dam during pregnancy, particularly at D16, in line with previous observations of obese pregnant women with and without GDM (Uebel *et al*. 2014). Plasma triglycerides, weight of the maternal liver and the liver‐derived hormone, IGF1, in the circulation were all reduced in HFHS dams at D16. Reduced IGF1 may have contributed to the increased adiposity and diminished lean mass of HFHS dams because maternal endocrine IGFI is known to reduce adiposity and increase amino acid uptake by skeletal muscle in pregnant guinea‐pigs (Sferruzzi‐Perri *et al*. 2006; Sferruzzi‐Perri *et al*. 2007 *a*,*b*). Hypotriglyceridaemia has been reported in fructose fed rats that also show increased adiposity during pregnancy (Jen *et al*. 1991) and, in both the present study and previous studies, this may reflect a redistribution of triglycerides with greater storage in maternal tissues and less in the circulation. Maternal liver weight was no longer reduced in HFHS dams at D19 in the present study, which paralleled an increase in hepatic activation of the growth‐promoting, MAPK signalling pathway by D19. Moreover, circulating hormones and metabolites had normalized in HFHS dams by this stage of pregnancy. Normalization of maternal glycaemia near term was also observed previously in pregnant rodents fed high‐fat or sucrose diets (Jen *et al*. 1991; Liang *et al*. 2010). In the present study, plasma NEFAs were lower in HFHS compared to control dams on D19, which is consistent with previous observations in near‐term pregnant mice fed high‐fat diets (Heerwagen *et al*. 2013). By contrast, in obese and GDM women NEFA concentrations are elevated or unchanged in late pregnancy (Catalano *et al*. 2002; Chen & Scholl, 2008).

Overall, the hyperinsulinaemia and fasting hyperglycaemia seen in the HFHS dams at D16 suggest maternal insulin resistance, although overt glucose intolerance was not observed until D19. Late gestational glucose intolerance has also been reported in mice fed a HFHS diet from before pregnancy; however, this was associated with fetal overgrowth (Rosario *et al*. 2015), which is similar to a subset of mothers with obesity. Impaired pancreatic β cell proliferation may have contributed to the glucose intolerance of HFHS dams. Proliferation of the β cells during mouse pregnancy depends on placental secretion of PRL/PL‐related proteins (Brelje *et al*. 1993). A HFHS diet reduced placental expression of *Prl/Pl*‐related genes on D16, which may have had consequences for β cell proliferation and the ability to respond to acute hyperglycemia (Huang *et al*. 2009). Further experiments measuring insulin concentrations during the glucose tolerance test are required to establish the relative importance of insulin secretion *vs*. insulin action with respect to the glucose intolerance induced by HFHS feeding.

**Figure 11:**
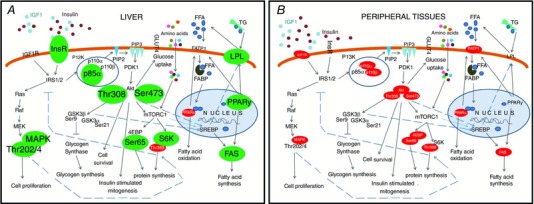
HFHS feeding differentially alters metabolic signalling pathways in maternal tissues in late pregnancy Schematic showing the effect of a HFHS diet on the expression of insulin signalling and lipid metabolism proteins in the liver (*A*) and the peripheral tissues (*B*, skeletal muscle and white adipose tissue) of the mouse dam in late pregnancy. Proteins that were upregulated are shown in large black font and in green/grey colour, whereas proteins that were downregulated are shown in small white font and in red/black colour. IGF1, insulin like growth factor 1; InsR, insulin receptor; IGF1R, insulin‐like growth factor receptor 1; IRS1, insulin receptor substrate 1; PI3K, phosphoinositide 3‐kinase; p85α, the regulatory subunit of PI3K; p110, the catalytic subunit of PI3K; PIP2, phosphatidylinositol 4,5‐biphosphate; PIP3, phosphatidylinositol 3,4,5‐phosphate; PDK1, phosphoinositol‐dependent kinase ‐1; Akt, protein kinase B; Thr308, threonine residue 308 of Akt; Ser473, serine residue 473 of Akt; GSK3, glycogen synthase kinase 3; Ser9, serine residue of GSK3β; Ser21, serine residue 21 of GSK3α; mTORC1, mechanistic target of rapamycin kinase complex 1; 4EBP, eukaryotic translation initiation factor 4E binding protein; Ser65, serine residue of 4EBP; S6K, ribosomal S6 kinase; Thr389, threonine residue of S6K; GLUT4, glucose transporter 4; FFA, free fatty acids; TG, triglycerides; FATP1, fatty acid transport protein 1; LPL, lipoprotein lipase; FABP, fatty acid binding protein; PPARα,γ, peroxisome proliferator‐activated receptors; SREBP, sterol regulatory element binding protein; FAS, fatty acid synthase; Ras, small GTPase protein; Raf, serine threonine protein kinase; MEK, mitogen extracellular signal regulated kinase; MAPK, mitogen activated protein kinase; Thr202, threonine residue of MAPK; Tyr204, tyrosine residue of MAPK. [Color figure can be viewed at wileyonlinelibrary.com]

At D19, a higher rate of glucose infusion was needed to maintain euglycaemia in HFHS than control dams. This was unexpected and suggested that HFHS feeding increased, rather than decreased, whole body insulin sensitivity of the dam. However, compared to controls, the maternal liver of HFHS dams was more sensitive to insulin at D19 because insulin more effectively suppressed EGP, which is consistent with the increased hepatic insulin receptor abundance. Even at D16, there was evidence of increased hepatic insulin signalling in HFHS dams because phosphoinositol 3‐kinase (PI3K)‐p85α expression and Akt activation (pAkt T308 at D16 and pAkt S473 at both D16 and D19) were enhanced compared to controls. Increased insulin signalling via the PI3K‐Akt pathway may have contributed to the observed fatty liver in HFHS‐fed dams because deletion of genes in this pathway protects against high‐fat diet‐induced hepatic steatosis in non‐pregnant mice (Leavens *et al*. 2009; Chattopadhyay *et al*. 2011). However, whole body glucose utilization was lower in both basal and hyperinsulinaemic states and glucose utilization during hyperinsulinaemia by the skeletal muscle was diminished in HFHS dams. Moreover, expression of several components of the insulin signalling pathway was downregulated in the skeletal muscle and WAT of HFHS dams on D16 and D19 and may have mediated the decreased expression of lipogenic markers in these tissues, in contrast to the hepatic data. In line with this, a diet high in sugar and/or fat reduced insulin sensitivity index and insulin responsiveness of isolated fat cells in late pregnant rats (Jen *et al*. 1991; Holemans *et al*. 2004) and decreased whole body insulin sensitivity and glucose uptake by the hindlimb in pregnant dogs (Moore *et al*. 2011; Woolcott *et al*. 2015). Furthermore, women with GDM displayed decreased whole body insulin sensitivity, skeletal muscle and adipose tissue insulin signalling and insulin‐stimulated glucose uptake during pregnancy (Garvey *et al*. 1993; Friedman *et al*. 1999; Shao *et al*. 2000; Catalano *et al*. 2002; Shao *et al*. 2002). Overall, the results of the present study indicate that HFHS feeding affects tissue insulin resistance in some, but not all, maternal tissues during mouse pregnancy (Fig. 11).

At D19, the liver of HFHS‐fed dams produced less glucose endogenously in both basal and hyperinsulinaemic states than controls. This was coupled to decreased hepatic expression of G6Pase, the rate‐limiting enzyme involved in gluconeogenesis and glycogenolysis. G6Pase is inhibited by insulin (van Schaftingen & Gerin, 2002) and its downregulation is consistent with increased hepatic insulin sensitivity and insulin receptor expression in HFHS dams. However, HFHS feeding did not cause an accumulation of glycogen in the maternal liver. This may have been secondary to the disrupted mTORC1 signalling observed in the liver of HFHS‐fed dams because the mTORC1 pathway regulates glycogen synthase, the enzyme responsible for producing glycogen (Wang *et al*. 2011). Reduced maternal glucogenesis is consistent with a higher dietary sugar intake and would explain the increased rate of glucose infusion required to maintain euglycaemia in the HFHS relative to control dams, especially given the large drain of glucose into the gravid uterus at D19. By decreasing glucose availability between feeding periods, the lower rates of gluconeogenesis could also have contributed to the restricted growth of the placenta and the lower body weights of the fetuses observed both in the present and previous studies at D16 (Sferruzzi‐Perri *et al*. 2013), when fetal growth is more dependent on glucose than amino acid availability (Coan *et al*. 2011).

The fetuses of the HFHS dams achieved a normal weight near term (both in the present and previous studies; Sferruzzi‐Perri *et al*. 2013). This occurred despite a smaller placenta and a reduced rate of glucose utilization, indicated by the lower p2DG accumulation in the HFHS fetuses at D19. These data suggest that fetuses in HFHS‐fed dams must use substrates other than glucose for growth and oxidative metabolism during late gestation. Indeed, previous studies have shown transfer of amino acids by the System A transporters, per unit surface area, is increased near term in HSHF‐fed dams (Sferruzzi‐Perri *et al*. 2013; Rosario *et al*. 2015). Moreover, placental FATP1 expression was upregulated by HFHS feeding on D19 (Sferruzzi‐Perri *et al*. 2013) suggesting that there may also be an increased materno‐placental supply of NEFAs to the HFHS fetuses. Fetal growth is correlated with placental amino acid but not glucose supply on D19 of mouse pregnancy (Coan *et al*. 2011) and an increased maternal supply of fats during pregnancy resulted in fatty liver in offspring (Elahi *et al*. 2009; McCurdy *et al*. 2009; Ashino *et al*. 2012; Alfaradhi *et al*. 2014). Collectively, these data show that the placental supply and fetal use of metabolic substrates are altered by a HFHS diet during pregnancy with potential consequences for fetal development and the programming of offspring metabolic phenotype (Samuelsson *et al*. 2008; Sferruzzi‐Perri *et al*. 2013). Further work is required to determine the relationship between maternal glucose‐insulin dynamics and feto‐placental glucose metabolism and growth in pregnancies in which the mother started to eat a HFHS diet prior to pregnancy.

In summary, HFHS feeding during pregnancy differentially altered insulin sensitivity of maternal tissues, with increased hepatic insulin sensitivity but decreased sensitivity of the skeletal muscle and WAT (Fig. 11). These changes were accompanied by increased adiposity and reduced glucose production and glucose tolerance of the dam in late pregnancy. They were also related to impaired nutrient partitioning between the mother and the gravid uterus and, hence, feto‐placental growth and metabolism. The changes in maternal metabolism, placental phenotype (Sferruzzi‐Perri *et al*. 2013) and fetal growth induced by HFHS feeding during pregnancy may explain, in part, the early life programming of adult disease by maternal obesity and obesogenic diets. They may also shed light on the mechanisms by which altered maternal‐fetal resource allocation during pregnancy increases the risk of poor health in the mother post‐partum.

## Additional information

### Competing interests

The authors have no competing interests.

### Author contributions

ANS‐P, BM, ALF and SEO designed the study. ANS‐P, ORV and BM time‐mated and fed the mice. BM and ANS‐P carried out the glucose tolerance tests. BM, DSF‐T, PV and ALF undertook the clamp studies. ANS‐P, ORV, BM and ALF collected the tissues. BM performed the Western blotting. BM, ANS‐P and ALF wrote the paper. All authors read and approved the final paper. ANS‐P is the guarantor of the paper.

### Funding

We are grateful to the Medical Research Council (MRC) for funding the research through a studentship to BM (MR/J500458/1), an *in vivo* skills award (MRC CORD G0600717) and the MRC Metabolic Diseases Unit (MC_UU_12012/4).
